# Immune Evasion Strategies of Relapsing Fever Spirochetes

**DOI:** 10.3389/fimmu.2020.01560

**Published:** 2020-07-23

**Authors:** Florian Röttgerding, Peter Kraiczy

**Affiliations:** Institute of Medical Microbiology and Infection Control, University Hospital of Frankfurt, Goethe University Frankfurt, Frankfurt, Germany

**Keywords:** spirochetes, *Borrelia*, relapsing fever, immune evasion, complement, antigenic variation, innate immunity, adaptive immunity

## Abstract

Relapsing fever (RF) is claimed a neglected arthropod-borne disease caused by a number of diverse human pathogenic *Borrelia* (*B*.) species. These RF borreliae are separated into the groups of tick-transmitted species including *B. duttonii, B. hermsii, B. parkeri, B. turicatae, B. hispanica, B. persica, B. caucasica*, and *B. myiamotoi*, and the louse-borne *Borrelia* species *B. recurrentis*. As typical blood-borne pathogens achieving high cell concentrations in human blood, RF borreliae (RFB) must outwit innate immunity, in particular complement as the first line of defense. One prominent strategy developed by RFB to evade innate immunity involves inactivation of complement by recruiting distinct complement regulatory proteins, e.g., C1 esterase inhibitor (C1-INH), C4b-binding protein (C4BP), factor H (FH), FH-like protein-1 (FHL-1), and factor H-related proteins FHR-1 and FHR-2, or binding of individual complement components and plasminogen, respectively. A number of multi-functional, complement and plasminogen-binding molecules from distinct *Borrelia* species have previously been identified and characterized, exhibiting considerable heterogeneity in their sequences, structures, gene localization, and their capacity to bind host-derived proteins. In addition, RFB possess a unique system of antigenic variation, allowing them to change the composition of surface-exposed variable major proteins, thus evading the acquired immune response of the human host. This review focuses on the current knowledge of the immune evasion strategies by RFB and highlights the role of complement-interfering and infection-associated molecules for the pathogenesis of RFB.

## Introduction

Relapsing fever (RF), an ectoparasite-borne bacterial disease caused by *Borrelia* species is characterized by recurrent episodes of high fever and spirochetemia in the blood of infected individuals (1–4). RF is a neglected and emerging bacterial disease in the Americas and certain African countries, especially in regions with a high incidence of infected argasid and ixodid ticks of the genus *Ornithodoros* and *Ixodes*, respectively, or the human body louse *Pediculus humanus* (2, 4). While soft tick-borne RF (STBRF) is mainly found along the West coast of North America and endemic in the temperate and tropical African territories, the occurrence of hard tick-borne RF (HTBRF) directly correlates with the distribution of ixodid ticks in the northern hemisphere (5, 6). In contrast, LBRF is geographically restricted to countries along the Horn of Africa, in particular Eritrea, Ethiopia, and South-Sudan (4). Despite its focal distribution, LBRF has the potential to dramatically re-emerge when sociodemographic factors such as war, famine, political turmoil, and precarious hygiene conditions in overcrowding camps change (7–11). Clinical signs of STBRF and LBRF appear abruptly between 2 and 18 days after infection with high fever, often accompanied by rigors, headache, chills, nausea, vomiting, myalgia, and diarrhea (2, 4). More severe clinical manifestations affect different organs such the liver (hepatosplenomegaly, liver dysfunction, hepatic failure), spleen (rupture), gastrointestinal tract (bleeding), lung (acute pulmonary edema, acute respiratory distress syndrome), heart (myocardial failure), and the central nervous system (meningism, facial paresis, vertigo, rigidity) (4). Concerning HTBRF, fever, headache, chills, arthralgia, fatigue, and malaise have been reported as the most common symptoms and severe neurological manifestations such as meningoencephalitis occur predominantly in immunocompromised patients (6, 12, 13). Like Lyme disease spirochetes, RFB exploit diverse immune evasion strategies to avoid recognition, and circumvent the innate and adaptive immune responses. Herein, we summarize the current knowledge of potential pathogenic factors identified in diverse RFB that counteract complement and humoral immune responses of the human host.

## The Complement System at a Glance

Complement operates as a first line of defense against intruding pathogens and consists of numerous fluid-phase and membrane-bound regulators, inhibitors and inactive precursor molecules, most of which act in concert upon activation to eliminate microbes (14). Just like a domino effect, the complement cascade can independently be activated through three distinct pathways: the alternative (AP), the classical (CP), and the lectin pathway (LP) (15, 16).

The AP is spontaneously activated by a so-called tick-over-process leading to the covalent attachment of activated C3b molecules to microbial surfaces and thus allowing a continuous monitoring for invasive human pathogens (opsonization) (17, 18). Activation of the CP is triggered by binding of C1q to surface-bound IgM or IgG clusters and the LP utilizes mannose-binding lectin (MBL), collectins, and ficolins to recognize carbohydrate moieties on bacterial cell surfaces (15, 16, 19–21). Upon activation, either the C3 convertases C3bBb (AP) or C4b2a (CP and LP) are formed, leading to a massive generation of activated C3b that covalently binds to foreign surfaces. Further downstream activation is driven by binding of C3b to the C3 convertases, resulting in the formation of the C5 convertases C3bBb3b or C4b2a3b. By engendering the C5 convertase, C5 is cleaved to C5a and C5b, which covalently binds to the target surface. This critical activation step initializes the terminal sequence (TS) and the assembly of the pore-forming membrane attack complex C5b-9 or MAC. The MAC, a ring-like structure, is composed of numerous C9 molecules, all of which integrate into the microbial membrane and ultimately cause lysis (22–24).

To protect self surfaces from excessive activation, complement is tightly controlled by a variety of soluble and cell-bound complement regulatory proteins (25). Concerning soluble regulators, the AP is regulated by factor H (FH) and the factor H-like protein 1 (FHL-1) (generated by alternative splicing of the *cfh* gene). Both regulators inactivate C3b to iC3b by acting as co-factors for factor I, thereby accelerating the decay of the membrane-bound C3 convertase. In contrast to FH and FHL-1, the factor H-related protein 1 (FHR-1) is supposed to be a regulator of the TS and appears to block the cleavage activity of the C5 convertases by inhibiting the generation of C5a (26). The role of the additional four FHR proteins in complement regulation is as yet unclear. Recent data provide some evidence that these proteins may enhance complement activation and, thus possess an opposite regulatory function compared to FH and FHL-1 (27). Initial activation of the CP and LP is controlled by C1 esterase inhibitor (C1-INH) by inactivation of the serine proteases C1r, C1s, MASP-1, and MASP-2, respectively. In addition, the downstream activation steps of the CP are terminated by binding of the C4b-binding protein (C4BP) to C4b. This soluble regulator acts as cofactor for the Factor I-mediated degradation of C4b. The TS is blocked by preventing the integration of the soluble preforming sC5b-9 complexes into the target membrane via vitronectin and clusterin (16).

## Recruitment of Complement Regulatory Proteins, an Efficient Strategy of RFB for Escaping Complement-Mediated Killing

Immediately upon entry into the mammalian host, RFB face complement as the first line of defense. However, the role of complement in spirochete clearance has controversially been discussed. It has been shown that IgM is able to efficiently kill *B. hermsii* in the course of bacteremia in infected C3- and C5-deficient mice by a complement-independent mechanism, while B cell-deficient mice showed very high loads of spirochetes in their blood (28, 29). These findings led to the assumption that innate immunity plays a subordinate role in the pathobiology of these pathogens. On the other hand, RFB produce complement-binding proteins, most of which operate on different activation levels to protect spirochetes from complement-mediated bacteriolysis (30–37). This includes proteins of *B. hermsii, B. parkeri, B. duttonii, B. miyamotoi*, and *B. recurrentis*, respectively. An overview of the characteristics of these suspected, infection-relevant surface proteins are given below.

### Inactivation of the Alternative Pathway by Binding of Complement Regulator FH

Acquisition of regulators of complement activation is one of the most common strategies exploited by many human pathogenic microorganisms to evade complement (38–40). At least seven FH-interacting proteins have been described among RFB species including BhCRASP-1 (FhbA1) of *B. hermsii* HS1 and FhbA2 (FhbA, FHBP19) of *B. hermsii* YOR, FHBP28 and BpcA of *B. parkeri*, CbiA of *B. miyamotoi*, and HcpA of *B. duttonii* and *B. recurrentis* (30, 31, 33, 34, 36, 37) (Table 1). All FH-interacting proteins have in common binding to the C-terminal domains implicating that the regulatory domains located at the N-terminus of FH are accessible to retain their Factor I-mediated C3b degradation activity (33, 34, 36, 37) (Table 1). Moreover, BhCRASP-1, HcpA, BpcA, and CbiA, respectively, facilitate complement resistance when ectopically produced in genetically manipulated spirochetes (gain-of-function strains) (33, 34, 36, 37).

**Table 1:** Characteristics of complement-binding proteins of relapsing fever borreliae.

**Complement binding protein**	**Genospecies**	**Strain**	**Gene localization**	**Gene locus**	**Gene name/ORF**	**Synonyms/other designations**	**Size (kDa)**	**Interacting complement regulator**	**Binding regions of complement regulator**	**Binding of complement component(s)**	**Interacting with specific host-derived proteins**	**Complement resistance (GOF)**	**Complement inhibition**	**References**
**STBRF**														
BhCRASP-1	*B. hermsii*	HS1	lp174	BHA008	*cspH*	FhbA1	21.5	FH FHR-1	SCR20	n.d.	Plasminogen	Yes	n.d.	(33, 36)
FhbA	*B. hermsii*	YOR HS1	lp174	n.d.^a^	*fhbA, bha008*	FHBP19 FhbA2	24	FH FHL-1	n.d.	n.d.	n.d.	n.d.	n.d.	(30, 31, 41)
FHBP28	*B. parkeri*	RML	n.d.	n.d.	n.d.	none	28	FH	n.d.	n.d.	n.d.	n.d.	n.d.	(30, 42)
BpcA	*B. parkeri*	RML	lp150	n.d.	n.d.	none	17	FH FHR-1	SCRs 19-20 SCRs 3-5	n.d.	Plasminogen	n.d.	n.d.	(36)
BtcA	*B. turicatae*	91E135	lp159	A7978_04350	n.d.	none	20.5	none	n.d.	n.d.	Plasminogen	n.d.	n.d.	(36)
BHA007	*B. hermsii*	HS1	lp174	BHA007	*bhA007*	none	39	C4BP	n.d.	n.d.	Fibronectin	n.d.	n.d.	(43)
**HTBRF**														
CbiA	*B. miyamotoi*	FR64b	lp70	CNO09_05070	*cbiA*	none	21	FH	SCRs 8-20 SCRs 15-20 SCRs 19-20	C3, C3b, C4b, C5	Plasminogen	Yes	CP, TS	(37, 44)
**LBRF**														
HcpA	*B. recurrentis*	A1 A17	lp124	n.d.	*hcpA*	none	21	FH FHR-1	SCRs 19-20 SCRs 3-5	C3, C3b, C4b	Plasminogen	Yes	TS	(34)
CihC	*B. recurrentis*	A1 A17	lp124	n.d.	*cihC*	none	40	C4BP C1-INH	n.d.	n.d.	Fibronectin	Yes	n.d.	(35)
CihC	*B. duttonii*	La	lp165	BDU_RSO4550	*cihC*	BDU_1	40	C4BP C1-INH	n.d.	n.d.	Fibronectin	Yes	n.d.	(35, 45)

*n.d.; not determined*.

*GOF, protein produced in a gain-of-function background; AP, alternative pathway; CP, classical pathway; TS, terminal sequence*.

a *Sequence of the fhbA gene could not be detected on lp200 of B. hermsii YOR and lp174 of B. hermsii HS1, respectively, by BLAST searches*.

Within a *Borrelia* species, the FH-binding proteins are highly conserved, exhibiting sequence identity values of >93% (32) but among RFB, the percentages are quite low (36–45%). Whether the lack of sequence similarity might account for a different fold, appears to be somewhat questionable, in particular in the light of missing three dimensional structures. Interestingly, at least four conserved motifs (LDxNQKQALIxF, LGN-KxKQFLQxLH, SFSSxNFxD, and LEQKKExAL) could be identified in all seven proteins, raising the possibility of a non-continuous FH-binding site. Further studies investigating variants of FhbA2, FHBP28, HcpA, and BpcA also provide evidence that multiple regions are involved in the interaction with FH (30, 34, 36, 41). Of importance, infection studies utilizing a *fhbA* deletion mutant demonstrated that FhbA2 is the only FH-binding protein of *B. hermsii* and the absence of FhbA did not have an impact on serum resistance or infectivity of spirochetes, indicating functionally redundant roles played by other complement-interacting proteins as discussed below (46).

### Inactivation of the Classical and Lectin Pathway by Binding of C1-INH and C4BP

**Figure 1:**
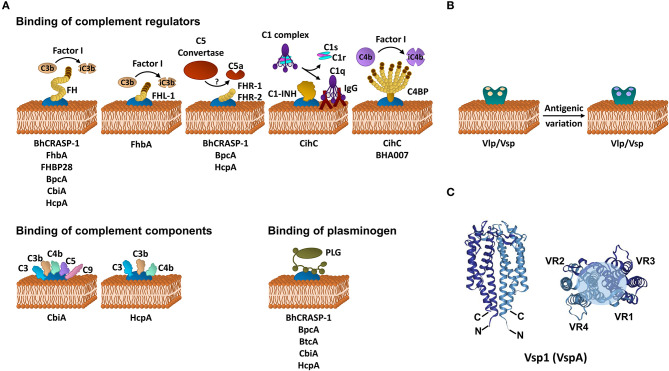
Immune evasion strategies of RFB. **(A)** Inhibition of complement by distinct borrelial proteins acting at certain levels of the activation cascade. **(B)** Immune evasion of RFB by multiphasic antigenic variation. **(C)** Schematic representation of the structure of the Vsp1 (VspA) dimer of *B. turicatae* [PDB 2GA0, adapted from (47)]. The monomeric units are represented in dark or light blue. The variable sequences are distributed within the second and third α-helices as well as all loop regions and summarize in variable region (VR) 1–4. The light blue circle at the top of the dimer indicates the region with the highest variability. FH, factor H; FHL-1, factor H-like protein 1; FHR, factor H-related protein; C4BP, C1-INH, C1 esterase inhibitor; C4b binding protein; iC3b, inactivated C3b; iC4b, inactivated C4b, IgG, immunoglobulin G.

To date, CihC of *B. recurrentis* is the soley protein displaying complement-inactivating properties on the CP and LP by binding to C1-INH and C4BP-binding protein (35). Orthologous proteins exhibiting sequence identities between 44 and 91% have been detected in *B. duttonii* Ly (BDU_1026), *B. hermsii* (BHA007), *B. turicatae* (BTA001), *B. parkeri* (BpA001), and *B. crocidurae* Achema and DOU (BCD_1370) but no homologous sequences could be found in LD spirochetes (42, 43). Functional analyses revealed that, like FH, C4BP bound to the borrelial surface retained its complement-inhibitory activity for factor I-mediated C4b degradation, thus targeting activation of the CP and LP (Figure 1A, Table 1). Previously, Meri et al. also demonstrated inactivation of the CP by binding of functional active C4BP to the surface of *B. recurrentis* and *B. duttonii* (45). In addition, CihC also promotes termination of the CP at the initial activation steps by binding of C1-INH, indicating that this borrelial molecule displays multi-functional complement-inhibitory properties. Deletions at the N- and C-terminus of CihC did not abrogate binding of C4BP or C1-INH leading to the assumption that central regions might be responsible for binding (Table 1). A crucial role of CihC in mediating serum resistance of RFB was evidenced by employing *cihC*-expressing gain-of-function strains which displayed a resistant phenotype upon serum challenge (35). In contrast to CihC, the BHA007 protein of *B. hermsii* only bound C4BP but not C1-INH (42). Owing to their functional properties to interact with fibronectin, these molecules have generically been named as “fibronectin-binding proteins” and clustered together with the fibronectin-binding BBK32 protein of Lyme disease spirochetes (42). Despite their low sequence similarity, the finding that BBK32 confers bloodstream survival of spirochetes (48) supports the possibility that CihC orthologs might also play a role during infection of the human host. Concerning CP inactivation, CbiA of *B. miyamotoi* has previously been shown to strongly inhibit activation of the CP, independently from interaction with C4BP by a yet unknown mechanism (37) (Figure 1A, Table 1). It is tempting to speculate whether binding of C4b to CbiA restricts downstream activation of the CP by terminating formation of the C3 convertase (37).

### Inhibition of the Terminal Sequence and MAC Assembly

Terminating the final activation steps by binding to pore-forming complexes or late complement components negatively affects assembling of the MAC as demonstrated for CbiA and HcpA (37) (Figure 1A, Table 1). In particular, CbiA strongly inhibits the TS, probably through the binding of C5 and C9 whereas HcpA moderately influences complement on this level and BpcA and BtcA, respectively, did not have an impact at all. Interference with the TS enhance the process of complement inactivation mediated by distinct outer surface proteins.

### Inactivation of Complement by Acquisition of Plasminogen

Distinct complement-interacting proteins including BhCRASP-1, HcpA, and CbiA exhibit multiple binding specificities to host-derived fluid phase proteins such as plasminogen (33, 34, 36, 44) (Figure 1A, Table 1). Plasminogen is known to bind to C3, C3b, C3d, and C5 and upon activation to plasmin, C3 and C5 degradation takes place (49). Plasminogen is also able to enhance Factor I-mediated C3b degradation in the presence of FH (49). Previous studies demonstrated that plasmin(ogen) bound to *B. hermsii* HS1, *B. recurrentis* A1, and *B. parkeri* decreases the amount of C3b molecules deposited on the borrelial surface (33, 34, 36) or lead to degradation of C3b when purified HcpA, BpcA, and CbiA, respectively, have been employed (34, 36, 44). Thus, degradation of C3 and C5 appears an additional strategy of RFB to successful overcome host immune defenses.

### Direct Interaction With Individual Complement Components

HcpA and CbiA also bind to some extent to individual complement components, namely C3, C3b, C4, and C4b, respectively, as well as C5 (CbiA) though the relevance of these interactions on complement inactivation require further investigation (37) (Figure 1A, Table 1).

In conclusion, these findings suggest an involvement of these molecules in immune evasion in particular as the inactivation of the key complement component C3b is thought to be an efficient instrument for bacterial survival and may account for the extraordinary pathogenesis of RFB in the human host.

## Antigenic Variation, a Powerful Mechanism of RFB to Escape Immune Avoidance

To evade clearance by the humoral immune response of the human host, RFB are capable of producing a bulk repertoire of antigenically distinct serotypes in a given cell population by a genetically driven process termed antigenic variation (50). In their pioneering work, Barbour and Stoenner revealed that the phenomenon of serotype switching is a spontaneous, reversible, and multiphasic process, creating outer surface proteins that bear serotype-specific epitopes (50) (Figure 1B). These immunodominant, variable major lipoproteins (Vmps) are divided into two different, highly polymorphic protein families: the variable small proteins, Vsp (~20 kDa) and the variable large proteins, Vlp (~36 kDa) that are subdivided in additional four subfamilies: α, β, γ, and δ (50–52). Apparently, the molecular mechanism of antigenic variation is not a subject to a process that is under pressure of the local environment, host factors or the host immune system. Recognizing that multiple serotypes arise from a single cell, thus, theoretically plenty of variants can be generated by producing highly diverse sets of Vlps and Vmps during infection. Previous studies revealed that 60–70 antigenically distinct variants of *B. hermsii* could arise during mammalian infection (52). Such a considerable diversity is achieved by multiple rounds of genetic rearrangements of the Vmp encoding genes including (i) non-reciprocal recombination of silent or archival *vmp* genes with an active, transcribed *vmp* gene (gene conversion), (ii) intramolecular DNA rearrangement, and (iii) switching of the expression site resulting in a modification of the transcript (53, 54). The mechanism of intermolecular recombination also appears to take place in the Old World RFB *B. duttonii* (55). Variable antigen genes encoding for Vlps and Vsps have also been detected in *B. turicatae, B. crocidurae, B. duttonii* (56, 57), *B. miyamotoi* (58, 59), and *B. recurrentis* (60, 61). Genetically, the silent or archival *vmp* genes are dispersed on different linear plasmids of 28–53 kb whereas an active, promoter-driven expression locus is found only on a single plasmid (53, 61). Such an active *vlp* or *vsp* gene can be exchanged by any archival or silent *vlp* and *vsp* gene but the frequency of replacement differs between these genes (62). The unceasing exchange of *vmp* genes will undoubtedly generate numerous polymorphic Vmps, allowing spirochetes to remain one step ahead of the adaptive immune response and thereby successfully evade the host's defenses. In a study using genetically modified *B. hermsii* cells that lack the ability to undergo antigenic variation, Raffel et al. clearly demonstrated that Vmps are required for inducing a high spirochetemia in the blood and for causing a relapse in infected mice whereby colonization of the ticks by these attenuated spirochetes remains unaffected (63). Interestingly, Vmp-lacking cells showed a reduce fitness compared to the WT and reconstituted spirochetes.

Crystal structure refinements of Vsp and Vlp revealed a similar fold for both groups of proteins which are predominately composed of a 2-fold-symmetric dimer. Each monomeric unit consists of four α-helical bundles connected by two loop regions (47, 64) (Figure 1C). The N-terminus is anchored in the spirochetal membrane while the flexible C-terminus is folded back and oriented closely to the N-terminus. The variable loop regions are exposed to the environment and serve as ligands for antibodies. Interestingly, the most conserved regions are oriented to the outside of the protein known to be targets for anti-Vmp antibodies elicited during infection. Of note, OspC, the major outer surface protein of *B. burgdorferi* is phylogenetically and structurally related, and shares a common helical fold to the Vsps suggesting that these proteins might display similar roles in immune evasion (47, 65).

## Concluding Remarks

Over the last decades, a number of complement-interacting molecules have been described, all of which touch the first line of host defense in certain ways by obstructing activation of complement. In synergy with the antigenic variation system, RFB are able to repeatedly circumvent both, the innate immune system as well as the acquired immune response. Understanding the molecular principles of how these molecules interfere with innate immunity may pave the way for developing new therapeutics for the treatment of RF patients or patients suffering from complement deficiencies, and might even serve as preventive measures for infectious diseases in general. Surface-exposed molecules may also be part of a new vaccine or can be used for the generation of novel immunoassays (32). Undoubtedly, future studies should unravel important questions addressing the role of functionally redundant, anti-complement proteins in the pathogenesis of these new-emerging pathogens.

## Author Contributions

FR and PK wrote the manuscript and prepared the figure and table. All authors contributed to the article and approved the submitted version.

## Conflict of Interest

The authors declare that the research was conducted in the absence of any commercial or financial relationships that could be construed as a potential conflict of interest.

## Abbreviations

BpcA: *B. parkeri* complement regulator-binding protein A
BtcA: *B. turicatae* plasminogen-binding protein
CbiA: complement binding and inhibitory protein A
CihC: complement inhibition via C4BP
C1-INH: C1 esterase inhibitor
C4BP: C4b binding protein
FhbA: FH-binding protein A
FH: Factor H
FHL-1: FH-like protein-1
FHR: FH-related protein
HcpA: human complement regulator and plasminogen-binding protein A
HTBRF: hard tick-borne relapsing fever
GAG: glycosaminoglycans
LBRF: louse-borne relapsing fever
MAC: membrane attack complex
RCA: regulators of complement activation
RF: relapsing fever
RFB: relapsing fever borreliae
SCR: short consensus repeats
STBRF: soft tick-borne relapsing fever
Vlp: variable large protein
Vmp: variable major protein
Vsp: variable small protein.
